# T95. PREVALENCE AND CONSEQUENCES OF CARDIAC AUTONOMIC DYSFUNCTION (CADF) IN 112 UNMEDICATED PATIENTS WITH SCHIZOPHRENIA

**DOI:** 10.1093/schbul/sby016.371

**Published:** 2018-04-01

**Authors:** Karl Bär, Andy Schumann, Alexander Refisch, Steffen Schulz, Andreas Voss, Berend Malchow

**Affiliations:** 1 University of Jena; 2 Ludwig Maximilian University

## Abstract

**Background:**

A shortened life expectancy of about 15–20 years in patients with schizophrenia (SZ) is very well documented. Cardiovascular disease has been identified as one of the leading causes of premature death. Beyond the effects accounted for by lifestyle and antipsychotic medications, several lines of evidence indicate a shared underlying pathophysiology between both schizophrenia and cardiovascular disease. The most obvious link lies in the relation between cardiac autonomic dysfunction (CADF) and the development of cardiovascular diseases. CADF has been extensively described in patients with schizophrenia, with main features like an increased heart rate at rest, reduced baroreflex sensitivity or increased variability of the QT interval. However, the definite influence of autonomic dysfunction for reduced life expectancy is still unknown. Thus, one has to identify patients at increased risk. Therefore, we established a scoring system based on heart rate variability (HRV)-measures from unmedicated SZ patients to quantify autonomic changes associated with an assumed cardiac risk profile.

**Methods:**

Autonomic measures were obtained from electrocardiogram recordings at rest in 112 unmedicated SZ patients and 112 age and gender matched healthy controls (HC). A rating score was obtained by relating 13 different, independent heart rate variability indices from every SZ patient to the 1st, 1.5th and 2nd standard deviations (SD) of the HC sample. According to the total amount of rating points, every SZ patient was classified into a corresponding subgroup of cardiac autonomic dysfunction (< 4 points = absent, 4 – 13 points = moderate, > 13 points = severe CADF). The selected HRV parameters contain different information of autonomic system modulation and have been proven very reliable in clinical research. Besides, symptom severity (Positive and negative syndrome scale) were determined as well as cardiac risk markers like BMI, smoking habits and physical activity.

**Results:**

Severe CADF was present in 29% of the tested unmedicated SZ patients, whereas moderate CADF was present in 44% and absent in 27% of the cases. Therefore, about one third of the patients revealed severe cardiac autonomic changes correlating with a potential risk for cardiovascular events. Only 27 % of the SZ patients showed no pathological findings. Patients with severe CADF showed significantly higher heart rates at rest when compared to patients with moderate CADF [F(2,109) = 23,089; p < 0,001]. The ratio of Low Frequency/High Frequency was also significantly higher in SZ patients with severe CADF compared to moderate CADF [F(2,109) = 38,321; p < 0,001] which points to a shift of the autonomic balance to sympathetic modulation. The three subgroups of SZ patients did not differ significantly in terms of symptoms, BMI, smoking habits or physical activity. SZ patients with severe CADF had a significantly longer duration of the illness [F(2,109) = 12,810; p < 0,001].

**Discussion:**

This study demonstrated that almost one third of unmedicated SZ patients show severe CADF. The close relation between CADF and the development of cardiac diseases or arrhythmias is well described. Therefore, these patients might need close cardiological follow-up appointments to reduce the likelihood of sudden cardiac death. In addition, the definite relation between the degree of autonomic dysfunction and the potential risk of cardiovascular events needs to be investigated in this patient population prospectively. Future studies need to design interventional strategies for everyday clinical settings to improve physical health and quality of life.

